# Efficacy of interventions for amblyopia: a systematic review and network meta-analysis

**DOI:** 10.1186/s12886-020-01442-9

**Published:** 2020-05-25

**Authors:** Yonghong Li, Huan Sun, Xiaojuan Zhu, Yana Su, Tianqi Yu, Xinyu Wu, Xiaoqin Zhou, Li Jing

**Affiliations:** 1 Department of Evidence-Based Medicine and Clinical Epidemiology, West China Hospital, Sichuan University, No.37 Guoxue Alley, Chengdu, 610041 China; 2 Mental Health Center and Psychiatric Laboratory, the State Key Laboratory of Biotherapy, West China Hospital of Sichuan University, No.37 Guoxue Alley, Chengdu, 610041 China; 3 Department of Ophthalmology, West China Hospital, Sichuan University, No.37 Guoxue Alley, Chengdu, 610041 China; 4 Department of Clinical Research Management, West China Hospital, Sichuan University, No.37 Guoxue Alley, Chengdu, 610041 China

**Keywords:** Amblyopia, Spectacles, Patching, Atropine, Optical penalization, Binocular, Meta-analysis

## Abstract

**Background:**

Many treatments are currently available for amblyopic patients; although, the comparative efficacy of these therapies is unclear. We conducted a systematic review and network meta-analysis (NMA) to establish the relative efficacy of these treatments for amblyopia.

**Methods:**

Electronic databases (MEDLINE, EMBASE, Cochrane Library) were systematically searched from inception to Sep. 2019. Only Randomized clinical trials comparing any two or three of the following treatments were included: refractive correction (spectacles alone), patching of 2 h per day (patch 2H), patch 6H, patch 12H, patch 2H + near activities (N), patch 2H + distant activities (D), atropine (Atr) daily, Atr weekly, Atr weekly + plano lens over the sound eye (Plano), optical penalization and binocular therapy. The reviewers independently extracted the data according to the PRISMA guidelines; assessed study quality by Cochrane risk-of-bias tool for randomized trials. The primary outcome measure was the change in best-corrected visual acuity (BCVA) expressed as log MAR lines. Direct comparisons and a Bayesian meta-analysis were performed to synthesize data.

**Results:**

Twenty-three studies with 3279 patients were included. In the network meta-analysis, optical penalization was the least effective of all the treatments for the change of visual acuity, spectacles (mean difference [MD], 2.9 Log MAR lines; 95% credibility interval [CrI], 1.8–4.0), patch 2H (MD, 3.3; 95% CrI, 2.3–4.3), patch 6H (MD, 3.6; 95% CrI, 2.6–4.6), patch 12H (MD, 3.4; 95% CrI, 2.3–4.5), patch 2H + N (MD, 3.7; 95% CrI, 2.5–5.0), patch 2H + D (MD, 3.5; 95% CrI, 2.1–5.0), Atr daily (MD, 3.2; 95% CrI, 2.2–4.3), Atr weekly (MD, 3.2; 95% CrI, 2.2–4.3), Atr weekly + Plano (MD, 3.7; 95% CrI, 2.7–4.7), binocular therapy (MD, 3.1; 95% CrI, 2.0–4.2). The patch 6H and patch 2H + N were better than spectacles ([MD, 0.73; 95% Crl, 0.10–1.40]; [MD, 0.84; 95% CrI, 0.19–1.50]).

**Conclusions:**

The NMA indicated that the efficacy of the most of the examined treatment modalities for amblyopia were comparable, with no significant difference. Further high quality randomized controlled trials are required to determine their efficacy and acceptability.

**Systematic review registration:**

CRD42019119843.

## Background

Amblyopia is a type of neurodevelopmental disorder that constitute a largest threat to the vision of children. The prevalence of amblyopia is estimated at 1–5% in different areas and studies [1–3]. And amblyopia can lead to permanently reduced vision if not treated properly. Based on the current evidence, amblyopia is mainly caused by uncorrected refractive error, strabismus, cataract and ptosis [4]. There is a plethora of visual function deficits of the amblyopic individual that could affect learning, daily activities and psychological state of affected children [5, 6]. Therefore, it is essential to get the best treatment during the critical period to avoid severe consequences later on due to this disorder.

More than ten therapeutic regimens have been used to treat amblyopia in clinics based on the theory of visual stimulation [4, 7–11]. Among them, conventional patching is still mainly used in clinical settings for its long-term, relative safety. However, the patching regimen usually depends on the ophthalmologists’ clinical impressions, training, and observations [7]. In addition, recent studies found that most of the adverse implications are caused by the treatment rather than the condition itself; patching is more likely to impact psycho-social and quality of life both for the child and the family [5, 12]. Innovative methods including pharmacological penalization and the binocular approach using virtual reality software and devices are not widely accepted and used currently, even though some studies have shown that these treatments are not less effective than patching [7]. To achieve better outcomes in both vision improvement and in reduction of adverse reactions, it is important to determine more clearly the effects of these different treatment paradigms.

Not all treatments have been directly compared and many randomized controlled trials (RCTs) and meta-analyses showed no difference between the specific treatments compared [13–18]. Therefore, we provide an NMA that allows for both direct and indirect comparisons to further clarify the efficacy of current interventions for amblyopia.

## Methods

A study protocol was registered with PROSPERO (CRD42019119843). This review adheres to the PRISMA extension statement for network meta-analyses [19].

### Data sources and search strategy

The Ovid MEDLINE, Ovid EMBASE, Cochrane Central Register of Controlled Trials (CENTRAL) were systematically searched to include relevant studies published in English from inception to 1st Sep. 2019 (see Additional file 1). The Cochrane Database of Systematic Reviews and reference lists of published reviews were screened to identify additional relevant studies. The searches were independently performed by the researchers and disagreements were discussed and resolved by consensus.

### Eligibility criteria

Included were the RCTs that enrolled patients with strabismus, anisometropia, mixed and residual amblyopia. Trials that compared at least two of the following 11 interventions were eligible: refractive correction (spectacles alone), patch 2 h per day (2H), patch 6H, patch 12H, patch 2H + near activities (N), patch 2H + distant activities (D), atropine (Atr) daily, Atr weekly, Atr weekly + plano lens over the sound eye (Plano), optical penalization and binocular therapy. All participants wore spectacles if prescribed (the detailed therapeutic regimens were shown in Additional file 2).

Exclusion criteria were study designs different from this study, interventions different from this study such as Bangerter filters or Amblyz liquid crystal occlusion glasses, no specific intervention time and data unsuitable for meta-analysis.

### Data collection and outcome measures

Researchers independently screened articles and extracted the data according to the inclusion criteria and the data extraction form used. When the same population were involved in multiple study publications, only the primary report was included in the meta-analysis. The outcome measure was the improvement of best-corrected visual acuity (BCVA) in the amblyopic eye expressed as log MAR units. The BCVA was obtained by a study-certified examiner using the Early Treatment Diabetic Retinopathy Study (ETDRS) protocol or Amblyopia Treatment Study VA-testing (ATS-HOTV) protocol [20, 21].

The following data were extracted: first author, year of publication, baseline demographic characteristics (age, sex, visual acuity and types of amblyopia), interventions, sample sizes, duration of treatment as well as outcomes. In addition, the characteristics of the study design were extracted to assess the risk of bias within included studies.

### Risk of Bias assessment

The risk of bias of included studies was assessed independently by the authors according to the seven domains of Cochrane Risk of Bias Tool for randomized trials [22]: random sequence generation, allocation concealment, blinding of the participants and personnel, blinding of outcome assessment, incomplete outcome data, selective outcome reporting and other biases.

### Data synthesis and analysis

Pairwise meta-analysis for all direct comparisons were performed using the random-effects model by Review Manager 5.3. Thereafter, the network meta-analysis was conducted based on a Bayesian framework random-effects model. The process of model specification, priors setting, starting values selection of multiple chains were automatically completed through Gemtc package [23], by which the consistency model was selected. Markov Chain Monte Carlo (MCMC) simulation was computed by running four chains simultaneously with 10,000 iterations discarded and 40,000 iterations obtained finally to achieve stability. The model convergence was checked by Brooks-Gelman-Rubin diagnostic statistics with potential scale reduction factor (PSRF) less than 1.05 considered acceptable, and through inspection of trace plot and density plots [24].

The validity of indirect and mixed comparisons should consider three main assumptions: heterogeneity, inconsistency and transitivity. The global heterogeneity was assessed using Chi-square test and *I*^2^ statistic, with *I*^2^ values greater than 75% indicating substantial heterogeneity, and the heterogeneity factor (*τ*^2^) was also calculated. The researchers planned to assess the inconsistency for each comparison by the node-splitting approach with two-side *p*-values < 0.05 considered statistically significant [25]. For the transitivity, only the clinical and methodological comparability of included studies can be described while there are no statistical methods for testing.

The results of continuous outcomes were expressed as mean differences (MD) with 95% credibility intervals (CrI), and the outcomes were interpreted as significant when the 95% Crl excludes the null value. The network meta-analysis was conducted in R software (version 3.4.0) interfacing with JAGS (version 4.3.0) [23]. The ranking probabilities of all interventions were estimated and the surface under the cumulative ranking curve (SUCRA) was provided with Stata (version 13.0) [26] (analysis code is placed in Additional file 3).

Researchers planned three sensitivity analyses for the change of amblyopic BCVA by removing (1) highly heterogeneous studies that lead heterogeneity greater than 75%, (2) studies with residual amblyopia and (3) studies with patients beyond 13 years of age.

## Results

### Characteristics and risk of bias of the included studies

**Fig. 1:**
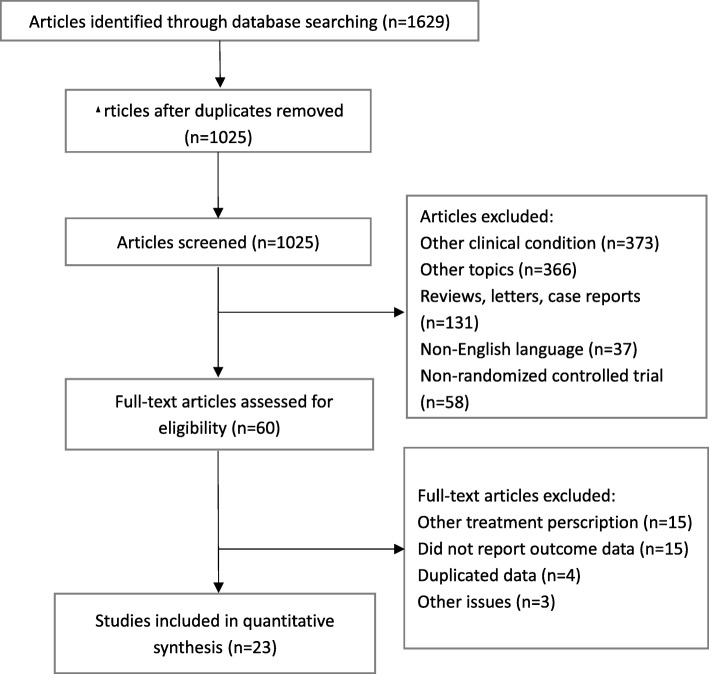
Flow diagram of literature screening

**Fig. 2:**
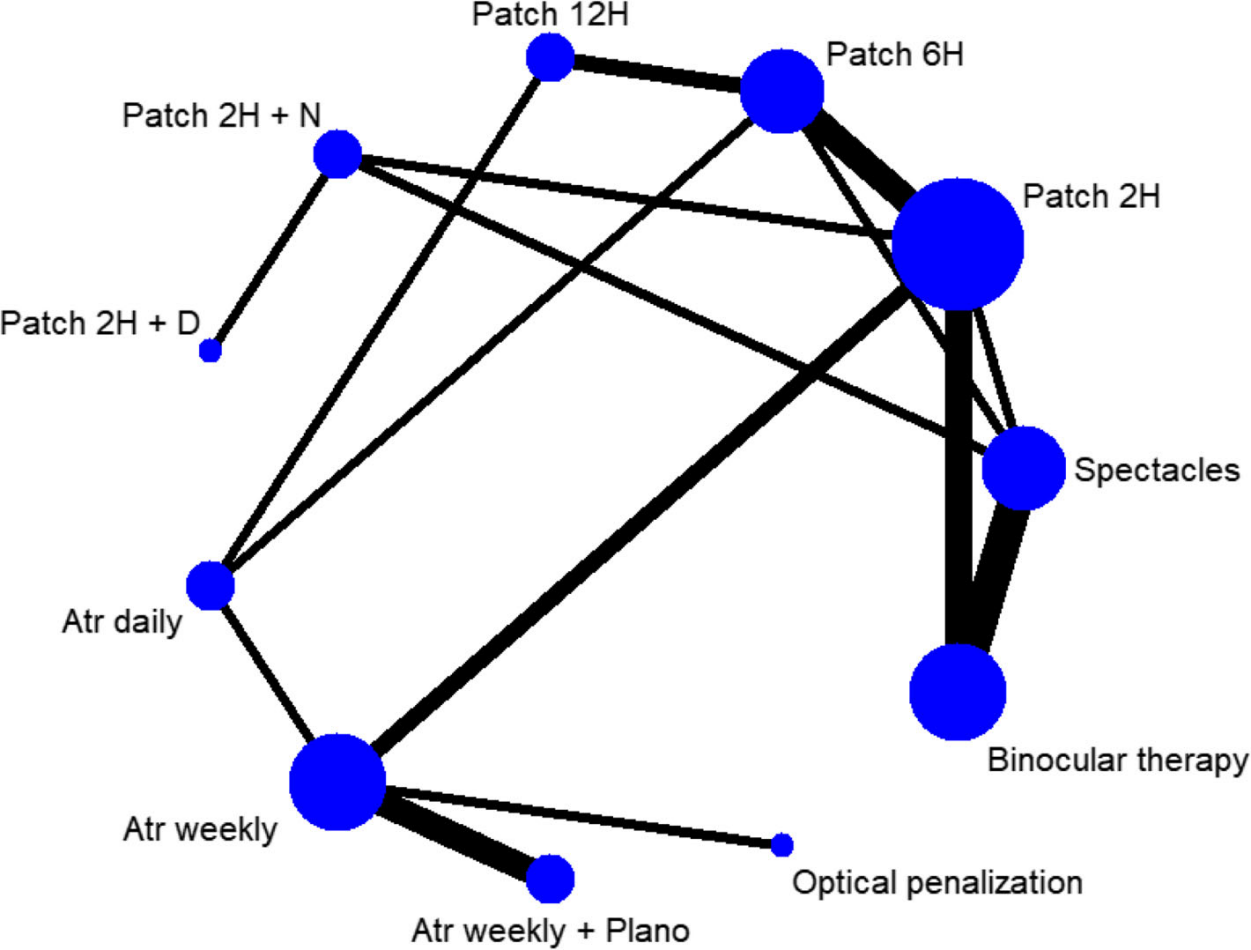
Network of eligible comparisons. The size of each circle represents the number of studies for the treatment, the line thickness of each connection denotes the number of studies investigating the comparison. H, hours per day; Atr, atropine; N, near activities; D, distant activities; Plano, plano lens over the sound eye

The PPSISMA diagram for systematic search and screening is shown in Fig. 1. Of 1629 relevant records, twenty-three studies with a total of 3279 patients were included in this network meta-analysis [27–49]. One (4.3%) of the studies contains two independent trials, one study was three-arm trial, and thirteen studies (56.5%) came from the Pediatric Eye Disease Investigator Group [50]. There were 15 comparisons among 11 different treatments (Fig. 2). Seventeen (70.8%) trials included patients under 10 years of age. All types of amblyopia were studied in seventeen (73.9%) studies, while one (4.3%) studies only included anisometropia, one (4.3%) study only included strabismus and two (8.6%) studies focused on residual amblyopia. The average follow-up time was 12.9 weeks (see Additional file 4).

We found low to moderate risk of bias in included studies, with 20 (86.9%) of studies reported adequate random sequence generation, 15 (65.2%) blinded outcome assessment and 20 (86.9%) reported all the randomized participants outcomes. However, most of the studies (86.9%) did not mention the allocation concealment; uncertainty about the reporting bias does exist because we had insufficient imformation to assess the risk of selective reporting bias in nine studies. In addition, some of the participants in studies had received prior treatments before the trial, which might increase the risk of selection bias (see Additional file 5).

### Direct meta-analysis

In direct comparisons, we only found that atropine weekly was associated with a better effect on the change of BCVA compared with optical penalization (MD, 3.22 log MAR lines; 95% CI, 2.72–3.72); the atropine weekly combined with a plano lens was more noticeable in improving visual acuity than that with atropine weekly alone (MD 0.44; 95% CI, 0.05–0.83) (Table 1).

**Table 1:** The results of direct meta-analysis

Comparisons	No. of Trials	No. of Patients	Mean Difference^**a**^ (95% CI)
Spectacles vs Patch 2H	1	35	− 0.51 (−1.70, 0.71)
Spectacles vs Patch 6H	1	35	−1.00 (−2.40, 0.38)
Spectacles vs Patch 2H + N	1	173	−0.60 (−1.40, 0.21)
Patch 2H vs Patch 2H + N	1	64	−1.00 (−2.20, 0.16)
Patch 6H vs Patch 12H	1	157	0.16 (−0.53, 0.85)
Patch 6H vs Atr daily	1	402	0.32 (−0.43, 1.10)
Patch 12H vs Atr daily	1	57	0.05 (−0.87, 0.93)
Patch 2H + N vs Patch 2H + D	1	392	0.20 (−0.14, 0.54)
Atr daily vs Atr weekly	1	168	−0.20 (−1.00, 0.61)
Atr weekly vs Optical penalization	1	63	**3.22 (2.72, 3.72)**
Spectacles vs Binocular therapy	4	341	−0.21 (−0.61, 0.13)
Patch 2H vs Patch 6H	3	379	−0.38 (− 0.91, 0.14)
Patch 2H vs Atr weekly	2	205	0.21 (−0.48, 0.89)
Patch 2H vs Binocular therapy	3	486	0.28 (−0.19, 0.75)
Atr weekly vs Atr weekly + Plano	2	300	**−0.44 (− 0.83, − 0.05)**

*H* hours per day, *Atr* atropine, *N* near activities, *D* distant activities, *Plano* plano lens over the sound eye. ^a^For the improvement of amblyopic BCVA, MD > 1 favored the treatment on the left side

### Network meta-analysis

**Fig. 3:**
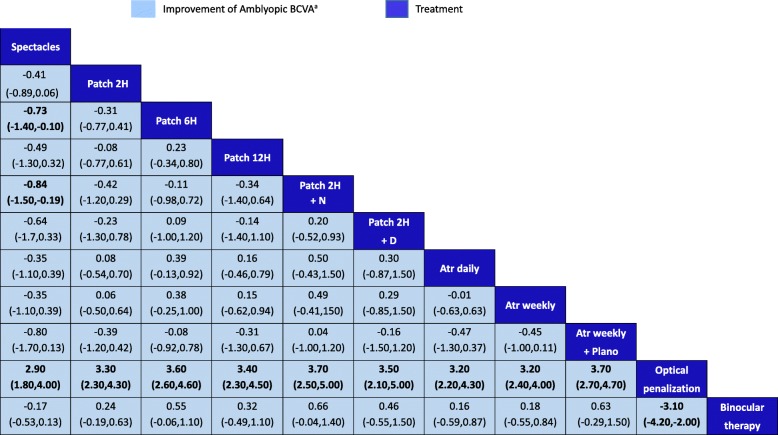
Results of network meta-analysis. ^a^For the improvement of amblyopic BCVA, MD > 1 favored the treatments on the left side of the Table. H, hours per day; Atr, atropine; N, near activities; D, distant activities; Plano, plano lens over the sound eye

In the network comparisons of the improvement of BCVA, the optical penalization was inferior to spectacles (MD, 2.90; 95% CrI, 1.80–4.00), patch 2H (MD, 3.30; 95% CrI, 2.30–4.30), patch 6H (MD, 3.60; 95% CrI, 2.60–4.60), patch 12H (MD, 3.40; 95% CrI, 2.30–4.50), patch 2H + N (MD, 3.70; 95% CrI, 2.50–5.00), patch 2H + D (MD, 3.50; 95% CrI, 2.10–5.00), Atr daily (MD, 3.20; 95% CrI, 2.20–4.30), Atr weekly (MD, 3.20; 95% CrI, 2.40–4.00), Atr weekly + Plano (MD, 3.70; 95% CrI, 2.70–4.70) and binocular therapy (MD, 3.10; 95% CrI, 2.00–4.20). When compared with spectacles, only patch 6H, and patch 2H + N showed better effectiveness with a MD of 0.73 (95% Crl, 0.10–1.40) and 0.84 (95% CrI, 0.19–1.50), respectively. No other statistical difference was found (Fig. 3). According to the rank probability and SUCRAs, patch 2H + N had the highest probability to be the best treatment which would result in greater improvements of BCVA. Thereafter, Atr weekly + Plano, patch 6H, and patch 2H + D were ranked in the next three positions (see Additional file 6).

There was no considerable heterogeneity in the direct meta-analysis except the comparison of spectacles versus binocular treatment (*I*^2^ = 95.57%). The global *I*^2^ was estimated to be zero. It was found that the direct and indirect results were consistent for the change of BCVA by using node-splitting approach (range of *p* values: 0.22–0.84) (see Additional file 7).

### Sensitivity analyses and subgroup analysis

In sensitivity analyses, we use spectacles as the reference intervention. After the removal of highly heterogeneous studies [43], studies including only residual amblyopia [35, 42] and those with patients over 13 years of age [40, 41], the effect value and conclusion changed slightly, whereas the ranking results of treatments in this NMA did not (Table 2). Due to the small number of studies, no subgroup analysis was carried out.

**Table 2:** The results of sensitivity analyses^a^

Treatment	Main analysis	Removal of high heterogeneity studies	Removal of residual amblyopia	Removal of patients over 13 years
Patch 2H	0.41 [− 0.06,0.89]	**0.32 [0.02,0.56]**	0.42 [− 0.03,0.90]	0.29 [−0.41,0.92]
Patch 6H	**0.73 [0.10,1.40]**	**0.64 [0.23,1.10]**	0.50 [−0.14,1.10]	0.57 [−0.35,1.40]
Patch 12H	0.49 [−0.32,1.30]	0.41 [−0.17,0.96]	0.29 [− 0.53,1.20]	0.34 [− 0.74,1.30]
Patch 2H + N	**0.84 [0.19,1.50]**	**0.75 [0.27,1.20]**	**0.83 [0.20,1.50]**	**0.82 [0.08,1.60]**
Patch 2H + D	0.64 [−0.33,1.70]	0.56 [−0.08,1.20]	0.63 [− 0.29,1.60]	0.62 [− 0.49,1.70]
Atr daily	0.34 [− 0.43,1.10]	0.25 [− 0.25,0.71]	0.17 [− 0.58,0.98]	0.18 [− 0.84,1.10]
Atr weekly	0.35 [− 0.39,1.10]	0.27 [− 0.32,0.73]	0.28 [− 0.43,1.00]	0.20 [− 0.76,1.10]
Atr weekly + Plano	0.80 [− 0.13,1.70]	0.72 [− 0.05,1.30]	0.70 [− 0.29,1.70]	0.67 [−0.48,1.70]
Optical penalization	**−2.90 [−4.00,-1.80]**	**−3.00 [− 3.70,-2.20]**	**−2.90 [− 4.00,-1.90]**	**−3.00 [− 4.30,-1.80]**
Binocular therapy	0.17 [− 0.13,0.53]	0.03 [− 0.17,0.20]	0.17 [−1.20,0.53]	0.18 [− 0.20,0.59]

*H* hours per day, *Atr* atropine, *N* near activities, *D* distant activities, *Plano* plano lens over the sound eye

^a^Data are mean differences (MD) and 95% Crl compared with refractive correction

## Discussions

Although many RCTs, pairwise meta-analysis and descriptive reviews have compared the therapeutic regimens for amblyopia [4, 51], it was not possible to have all regimens being compared head-to-head in a study owing to the large range of treatments. Current guidelines for the treatment of amblyopia are mostly based on the results of randomized controlled trials, since there was lack of high level evidence. Therefore, the researchers adopted the network meta-analysis techniques combined with latest RCTs to provide more guidance for the treatment of amblyopia.

Using data from twenty-four RCTs with 3279 participants, the results of direct comparisons showed that Atr weekly was worse than Atr weekly combined with plano lens over the sound eye, but better than optical penalization for the improvement of visual acuity, with a difference of 0.44 and 3.22 log MAR lines, respectively. According to network meta-analysis, all the studied interventions were found to be more effective than optical penalization. In addition, patch 6H, patch 2H + N were more effective than refractive correction with spectacles. The ranking of efficacy is as follows: patch 2H + N, Atr weekly + Plano, patch 6H, patch 2H + D, patch 12H, patch 2H, Atr weekly, Atr daily, binocular therapy, spectacles and optical penalization.

From historical research, three systematic reviews have been conducted to compare the conventional patching therapy with atropine penalization; both of the results suggested that atropine was as effective as patching in improving visual acuity [15, 52, 53]. In a head-to-head meta-analysis, there was no difference between part-time and full-time occlusion [14]. Moreover, Shotton (2008) concluded that spectacles alone is beneficial for unilateral amblyopia [54]. Taylor (2011) reported that patching appears to be more effective than spectacles while the benefit of adding near activities to patching is unproven [55]. However, it was found that most of the results were based on the descriptions of randomized controlled trials with no pooled analysis. Different from other studies is that we separated the interventions in greater detail and provided a global comparison as well as ranks of these treatments based on SUCRA values.

According to the ranking, patching regimens seem to be better than atropine except for the atropine weekly combined with a plano lens over the sound eye. Since the addition of plano lens is analogous to optical penalization or even patching, it is suggested that more studies are needed to determine the real difference between patching and atropine therapy. Consistent with previous studies [14], it was found that there was no significant difference between 2 h, 6 h, and 12 h of patching, while the addition of 1 h of activities (near or distant) to patching seemed to be more effective than patching alone. However, activities are usually used in combination with patching and there are no RCTs to evaluate the effect of activity treatment alone.

Binocular therapy is considered to be the most important development in the field of amblyopia for the past decade [8, 56]. To date, binocular therapy has only been compared with 2 h of patching and spectacles, and the present NMA result also showed that the efficacy of binocular treatment is not encouraging in improving visual acuity. Hence, using the binocular approach as a routine treatment for amblyopia is currently not recommended.

Another common studied treatment for the amblyopia is levodopa. However, this intervention was not included in this NMA due to the current levodopa treatment including a wide range of dose and duration [57]. Traditional Chinese acupuncture (TCA) could be a potential treatment for amblyopia by regulating neurotransmitters and neurotrophic factor in the visual system, and promoting the expression of genes related to visual plasticity [58]. Since there are few studies on acupuncture treatment and only anisometropia amblyopia was included in these studies, acupuncture was excluded from this study.

This review has several limitations. Firstly, the number of studies included are not enough to make a reliable estimate for the outcomes, several interventions had no direct comparisons and two interventions (patch 2H + D and optical penalization) were only studied in one trial. Secondly, there was a wider range in participants ages (3 to 20 years). Some studies reported only the age range or mean, thus we were unable to perform a subgroup analysis to determine the impact of age. Likewise, the data was insufficient to analyze the effects of amblyopia severity on treatment outcome. Thirdly, all the analyses are based on the treatment duration specified in trial design or estimated rather than the actual time of patching. Moreover, other efficacy outcomes and safety of interventions were not presented in this NMA due to the lack of such information and data in the studies concerned.

Despite the valuable data gathered on this subject, several aspects remain to be considered in future researches. First and foremost, in interpreting the results of any studies and designing new studies for amblyopia treatments, there needs to be an awareness of the potential biases including age, baseline refractive error, subtype and severity of amblyopia, previous treatment as well as study duration, since each may affect the efficacy of the treatment. It is also recommended to select a more clearly defined and narrow population for future studies. Another option to reveal the impact of these counfounding factors is to perform post-hoc analyses in studies. In addition, in amblyopia studies, there are practical barriers to randomization, blindness and obtaining complete and accurate data; it is also difficult to eliminate all forms of biases, specifically those caused by compliance. Thus, non-randomized studies with clearly defined inclusion criteria and clear protocol are also required in the future. On the basis of these sufficient and robust clinical trials, we can conduct more qualitative and quantitative reviews and provide high-level evidence for the treatment of amblyopia.

## Conclusions

Differences in clinical efficacy among various amblyopia treatments might exist but could not be readily confirmed from the available data. According to the rankings, traditional patching regimens are probably more effective than atropine, unless atropine is used in combination with suppression of the sound eye. And the addition of activities appears to enhance the effect of patching. Further larger high-quality clinical trials are warranted in order to establish their efficacy with a higher degree of credibility.

## Supplementary information

**Additional file 1.** Search strategy.

**Additional file 2.** Therapeutic regimens.

**Additional file 3.** Software code used.

**Additional file 4.** Baseline characteristics of included trials.

**Additional file 5.** Risk of bias assessment.

**Additional file 6.** Rank probability and SUCRAs.

**Additional file 7.** Inconsistency analysis.

## Data Availability

The datasets used and/or analyzed during the current study are available from the corresponding author on reasonable request.

## Abbreviations

NMA: Network meta-analysis
RCT: Randomized controlled trial
BCVA: Best corrected visual acuity
MD: Mean difference
Cl: Confidence interval
Crl: Credibility interval
SD: Standard difference
H: Hours per day
Atr: Atropine
N: Near activities
D: Distant activities
Plano: Plano lens over the sound eye
log MAR: Logarithm of minimal angle of resolution
TCA: Traditional Chinese acunpture
