# Supplementary material for: Efficacy of interventions for amblyopia: a systematic review and network meta-analysis
Source: BMC Ophthalmol. 2020 May 25;20:203. doi: 10.1186/s12886-020-01442-9 (PMC7249307; doi:10.1186/s12886-020-01442-9)
Supplement: Supplementary file 1 — Additional file 1. Search strategy. [file 12886_2020_1442_MOESM1_ESM.pdf]

Additional file 1:Search strategy

Medline(ovid)	EMBASE(ovid)	Cochrane Central Register of Controlled Trials
1randomized controlled trial.pt.<br>2 controlled clinical trial.pt.<br>3 random*.af.<br>4 placebo.af.<br>5 1or2or3or4<br>6 exp amblyopia/<br>7 amblyop*.af.<br>8 6or7<br>9 5and8<br>10 exp animals/<br>11 exp humans/<br>12 10 and 11<br>13 9and11<br>14 9and12<br>15 13or14	1 exp controlled clinical trial/<br>2 exp randomization/<br>3 randomized controlled trial/<br>4 double blind procedure/<br>5 single blind procedure/<br>6 random*.af.<br>7 placebo*.af.<br>8 or/1-7<br>9(animal or animal experiment).sh.<br>10 human.sh.<br>11 9 and 10<br>12 8 and10<br>13 8 and11<br>14 exp amblyopia/<br>15 amblyop*.af.<br>16 anopsi*.af.<br>17 14or15or16<br>18 12or13<br>19 17and18	1 MeSH descriptor: [Randomized Controlled Trial] explode all trees<br>2 MeSH descriptor: [Controlled Clinical Trial] explode all trees<br>3 random*.af.<br>4 trial:ti,ab,kw<br>5 1or2 or3 or 4<br>6 exp amblyopia/<br>7 amblyop*.af.<br>8 6 or 7<br>9 5 and 8
