# Supplementary material for: Efficacy of interventions for amblyopia: a systematic review and network meta-analysis
Source: BMC Ophthalmol. 2020 May 25;20:203. doi: 10.1186/s12886-020-01442-9 (PMC7249307; doi:10.1186/s12886-020-01442-9)
Supplement: Supplementary file 2 — Additional file 2. Therapeutic regimens. [file 12886_2020_1442_MOESM2_ESM.pdf]

Additional file 2: therapeutic regimens

Spectacles	Participants were just provided with spectacles and/or contact lenses if needed
Patch 2H	2 hours patching of the sound eye per day
Patch 6H	6 hours patching of the sound eye per day
Patch 12H	12 hours patching per day or full-time patching of the sound eye
Patch 2H + N	In addition to the patching, the child spend at least 1 hour doing near visual activities.
Patch 2H + D	In addition to the patching, the child spend at least 1 hour doing distance visual activities.
Atr daily	atropine 1% eye drops (per day)
Atr weekly	atropine 1% eye drops (Saturday and Sunday)
Atr weekly + Plano	atropine 1% eye drops (Saturday and Sunday) while wearing a plano lens over the sound eye
Optical penalization	positive defocus of the sound eye
Binocular therapy	1 hour per day of the binocular games on electronic devices

Abbreviations: *H* hours per day, *Atr* atropine, *N* near activities, *D* distant activities, *Plano* plano lens over the sound eye
