# Supplementary material for: Efficacy of interventions for amblyopia: a systematic review and network meta-analysis
Source: BMC Ophthalmol. 2020 May 25;20:203. doi: 10.1186/s12886-020-01442-9 (PMC7249307; doi:10.1186/s12886-020-01442-9)
Supplement: Supplementary file 3 — Additional file 3. Software code used. [file 12886_2020_1442_MOESM3_ESM.pdf]

Additional file 3: Software code used

**R software**	library(gemtc);library(coda);library(lattice);<br>network<-read.mtc.network("C:/ ");<br>plot(network)<br>summary(network)<br>mtc.anohe(network)<br>mtc.nodesplit(network)<br>model<-mtc.model(network, linearMode="random", type = "consistency", n.chain = 3);<br>results <-mtc.run(model, sampler ="JAGS", n.adapt = 10000, n.iter = 40000, thin = 1)<br>gelman.plot(results) #convergence<br>gelman.diag(results)<br>plot(results)<br>forest(relative.effect(results,"/"))<br>rank.probability(results)
**Stata software**	network setup mean sd n, study(studyid) trt(t)<br>network convert pairs<br>networkplot t1 t2<br>sucra nomv stats("C:/ effectivenwss.txt") rprob(effectiveness),rankog
