# Supplementary material for: Efficacy of interventions for amblyopia: a systematic review and network meta-analysis
Source: BMC Ophthalmol. 2020 May 25;20:203. doi: 10.1186/s12886-020-01442-9 (PMC7249307; doi:10.1186/s12886-020-01442-9)
Supplement: Supplementary file 4 — Additional file 4. Baseline characteristics of included trials. [file 12886_2020_1442_MOESM4_ESM.pdf]

Additional file 4: Baseline characteristics of included trials

Authors	Age, y	Type of Amblyopia			Grade of Severity[a]	Treatment[b]	Number of Patients	Baseline BCVA in the Amblyopic eye, LogMAR(SD)	Follow-up, w
		Stra	Anis	Mixd					
Glaser 2002	3-7		✓	✓	Moderate	Patch 6H	208	0.52(0.13)	24
						Atr daiy	194	0.53(0.14)	
Beck 2003	3-7	✓	✓	✓	Severe	Patch 6H + N	73	0.89(0.18)	16
						Patch 12H + N	84	0.90(0.18)	
Repka 2003	3-7	✓	✓	✓	Moderate	Patch 2H + N	92	0.48(0.10)	16
						Patch 6H + N	89	0.48(0.11)	
Pediatric 2004	3-7	✓	✓	✓	Moderate	Atr daiy	77	0.46(0.10)	16
						Atr weekly	83	0.46(0.11)	
Pediatric 2005	3-7	✓	✓	✓	Both	Patch 2H + N	32	0.67(0.31)	4
						Patch 2H	32	0.66(0.26)	
Pediatric 2006	3-7	✓	✓	✓	Both	Patch 2H + N	85	0.56(0.20)	5
						Spectacles	88	0.55(0.25)	
Stewart 2007	5.6(1.5)[c]	✓	✓	✓	Both	Patch 6H	40	0.45 (0.30)	18
						Patch 12H	40	0.44 (0.30)	
Tejedor 2007	2-10	✓	✓	✓	Moderate to mild	Atr weekly	31	0.43 (0.12)	6
						Optical penalization	32	0.44 (0.11)	
Pediatric 2008	3-7	✓	✓	✓	Both	Patch 2H + D	201	0.58 (0.22)	17
						Patch 2H + N	191	0.56 (0.23)	
Cotter 2009	3-7	✓	✓	✓	Moderate	Atr weekly	84	0.47 (0.12)	18
						Art weekly + Plano	88	0.50 (0.13)	
Repka 2009	3-6	✓	✓	✓	Severe	Atr weekly	24	0.99 (0.16)	18
						Atr weekly + Plano	31	1.01 (0.20)	
Repka 2009	7-13	✓	✓	✓	Severe	Atr weekly	20	0.95 (0.15)	17
						Patch 2H	13	0.95 (0.10)	
Pediatric 2013	3-8				Residual	Patch 2H	82	0.43 (0.15)	10
						Patch 6H	82	0.45 (0.16)	
Holmes 2016	5-12	✓	✓	✓	Severe to moderate	Binocular therapy	177	0.51 (0.17)	16
						Patch 2H	186	0.48 (0.17)	
Kelly 2016	4-10	✓	✓	✓	Moderate	Binocular therapy	13	0.48(0.14)	2
						Patch 2H	14		
Pediatric 2015	3-8				Residual	Atr weekly	40	0.48(0.19)	10
						Art weekly + Plano	33	0.48(0.16)	
Awan 2005	< 8	✓		✓	Severe to moderate	Spectacles	18	0.59 (0.23)	12
						Patch 3H	17	0.63 (0.17)	
						Patch 6H	17	0.69 (0.22)	

(continued)

Authors	Age, y	Type of Amblyopia			Grade of Severity	Treatment	Number of Patients	Mean(SD) BCVA in the Amblyopic eye, LogMAR	Follow-up, mo
		Stra	Anis	Mixd					
Gao 2018	≥7	✓	✓	✓	Severe to moderate	Spectacles	50	0.51(0.18)	6
						Binocular therapy	57	0.53(0.16)	
Scheiman 2009	7-12	✓	✓	✓	Moderate	Atr weekly + N	88	61.7 (6.6)letters[d]	17
						Patch 2h + N	84	62.4 (5.7)letters	
Manh 2018	13-16	✓	✓	✓	Severe to moderate	Patch 2h	56	56.1 (9.7)	16
						Binocular therapy	39	58.8 (9.1)	
						Acupuncture	41	0.46 (0.16)	
Menon 2008	8-20		✓		Severe to moderate	Patch 12H	29	0.68(0.12)	24
						Atr daiy	28	0.67(0.17)	
Holmes 2019	7-12	✓	✓	✓	Severe to moderate	Spectacles	67	60.0 (7.8)letters	8
						Binocular therapy	67	59.1 (8.2)letters	
Rajavi 2016	3-10	✓	✓	✓	All	Patching	25	0.33 (0.17)	4
						Binocular + patching	25	0.34 (0.14)	
Herbison 2016	4-8	✓	✓	✓	All	Spectacles	24	0.50 (0.20)	10
						Binocular therapy	26	0.49 (0.17)	

Abbreviations: *y* years, *SD* standard difference, *w* week, *Stra* strabismus, *Anis* anisometropia, *H* hours per day, *Atr* atropine, *Plano* plano lens over the sound eye

[a] Grade according to visual acuity with mild amblyopia: > 20/40; moderate amblyopia: 20/40-20/100; severe amblyopia: 20/125-20/400

[b] All the treatments are based on spectacle correction

[c] Data are reported as average age with standard error

[d] Data are given as letter score, with a score of 85 letters approximating 20/20 visual acuity and 5 letters approximating 1 log MAR line of acuity
