# Supplementary material for: Efficacy of interventions for amblyopia: a systematic review and network meta-analysis
Source: BMC Ophthalmol. 2020 May 25;20:203. doi: 10.1186/s12886-020-01442-9 (PMC7249307; doi:10.1186/s12886-020-01442-9)

Additional file 5: Quality assessment of included studies

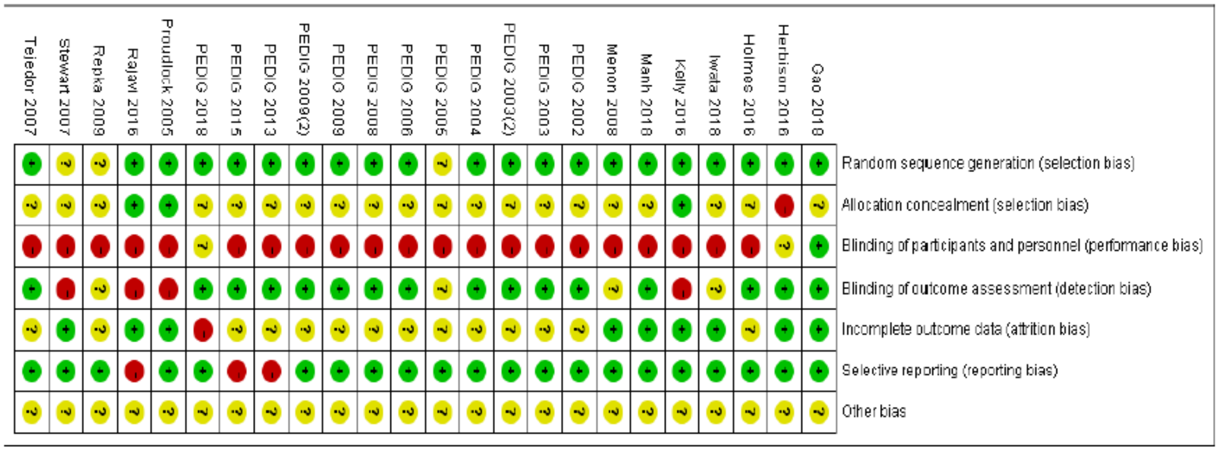

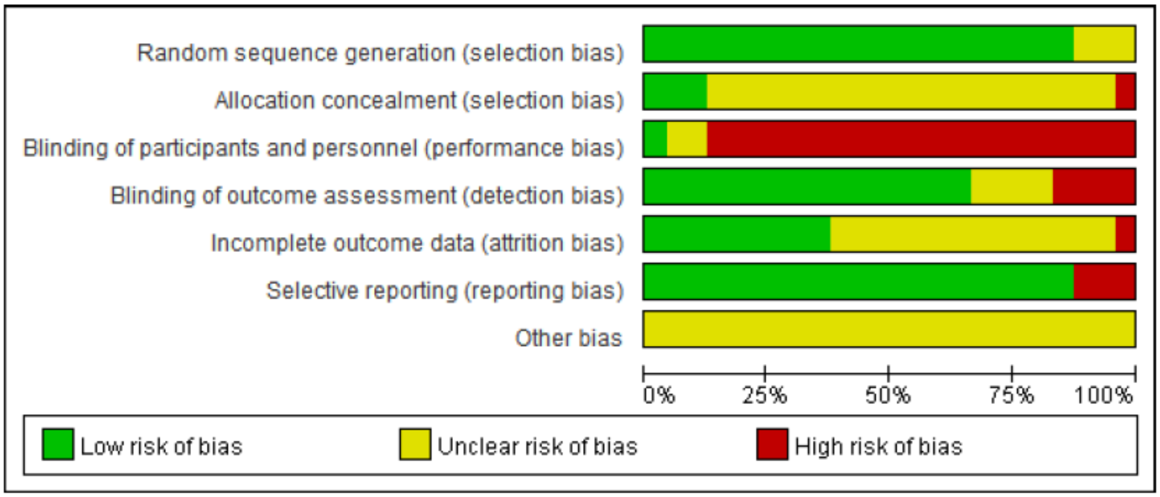

Supplement: Supplementary file 5 — Additional file 5. Risk of bias assessment. [file 12886_2020_1442_MOESM5_ESM.pdf]
