# Supplementary material for: Efficacy of interventions for amblyopia: a systematic review and network meta-analysis
Source: BMC Ophthalmol. 2020 May 25;20:203. doi: 10.1186/s12886-020-01442-9 (PMC7249307; doi:10.1186/s12886-020-01442-9)
Supplement: Supplementary file 6 — Additional file 6. Rank probability and SUCRAs. [file 12886_2020_1442_MOESM6_ESM.pdf]

Additional file 6: Rank probability and SUCRAs

	1	2	3	4	5	6	7	8	9	10	11
Spectacles	0.00	0.00	0.00	0.01	0.01	0.02	0.05	0.09	0.20	0.61	0.00
Patch 2H	0.00	0.01	0.05	0.12	0.21	0.24	0.20	0.13	0.02	0.01	0.00
Patch 6H	0.14	0.23	0.26	0.21	0.09	0.04	0.02	0.01	0.00	0.00	0.00
Patch 12H	0.05	0.09	0.12	0.15	0.15	0.12	0.11	0.09	0.07	0.05	0.00
Patch 2H + N	0.30	0.27	0.15	0.10	0.07	0.05	0.04	0.02	0.01	0.00	0.00
Patch 2H + D	0.16	0.18	0.13	0.10	0.08	0.07	0.07	0.08	0.06	0.07	0.00
Atr daily	0.01	0.02	0.05	0.09	0.13	0.16	0.17	0.17	0.12	0.10	0.00
Atr weekly	0.00	0.03	0.05	0.09	0.13	0.16	0.17	0.16	0.11	0.09	0.00
Atr weekly + Plano	0.34	0.16	0.17	0.11	0.08	0.05	0.04	0.03	0.02	0.01	0.00
Optical penalization	0.00	0.00	0.00	0.00	0.00	0.00	0.00	0.00	0.00	0.00	1.00
Binocular therapy	0.00	0.00	0.01	0.03	0.05	0.09	0.14	0.22	0.40	0.06	0.00

Abbreviations: *H* hours per day, *Atr* atropine, *N* near activities, *D* distant activities, *Plano* plano lens over the sound eye

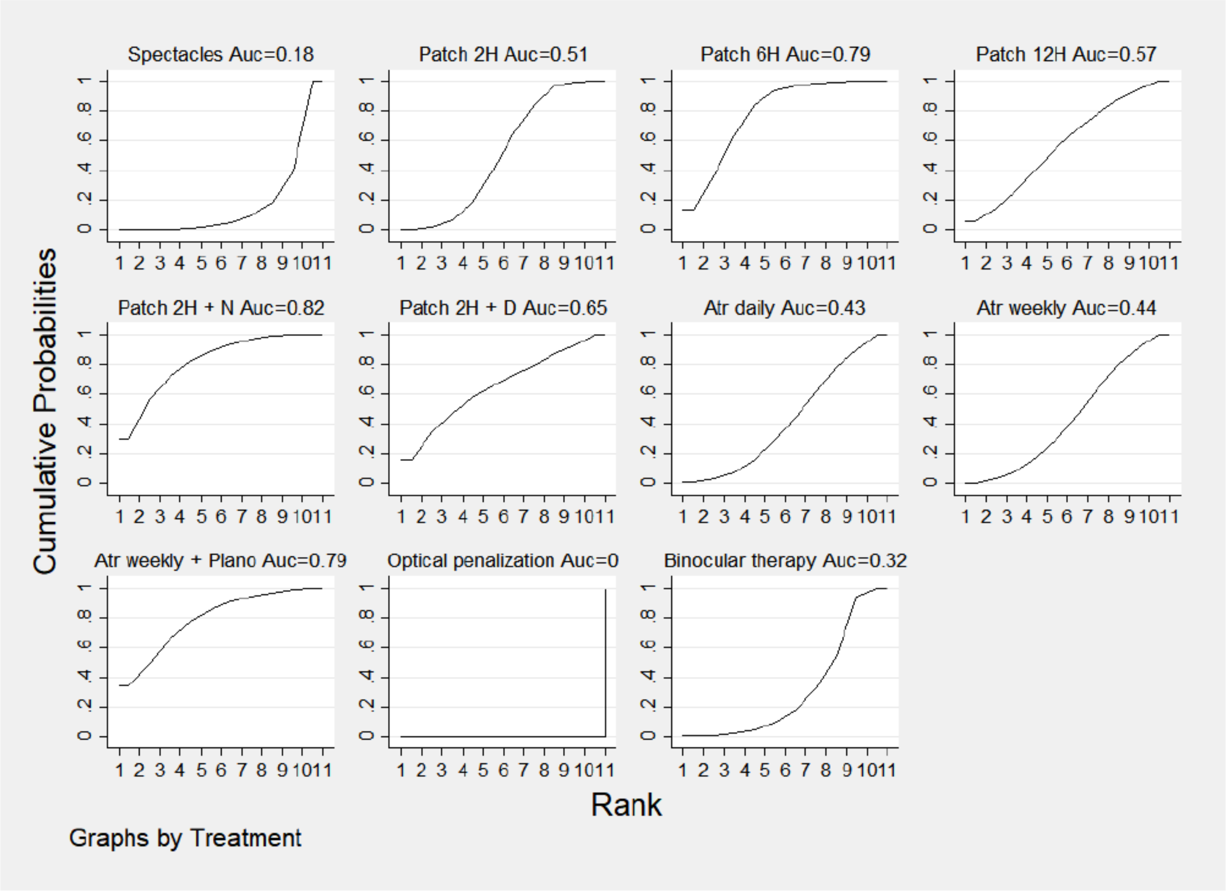

Abbreviations: *H* hours per day, *Atr* atropine, *N* near activities, *D* distant activities, *Plano* plano lens over the sound eye
