# Supplementary material for: Efficacy of interventions for amblyopia: a systematic review and network meta-analysis
Source: BMC Ophthalmol. 2020 May 25;20:203. doi: 10.1186/s12886-020-01442-9 (PMC7249307; doi:10.1186/s12886-020-01442-9)
Supplement: Supplementary file 7 — Additional file 7. Inconsistency analysis. [file 12886_2020_1442_MOESM7_ESM.pdf]

Additional file 7: Inconsistency test

Comparisons	Direct	Indirect	Network	P value
Patch 2H vs Spectacles	0.51 (-0.70, 1.70)	0.38 (-0.20, 0.94)	0.42 (-0.052, 0.90)	0.84
Patch 6H vs Spectacles	1.00 (-0.38, 2.40)	0.67 (-0.13, 1.40)	0.73 (0.11, 1.4)	0.67
Patch 2H + N vs Spectacles	0.60 (-0.21, 1.4)	1.50 (0.24, 2.8)	0.85 (0.19, 1.6)	0.23
Binocular vs Spectacles	-0.21 (-0.13, 0.61)	-0.09 (-1.1, 0.93)	0.17 (-0.13, 0.53)	0.56
Patch 6H vs Patch 2H	0.38 (-0.14, 0.91)	-0.13 (-1.4, 1.1)	0.32 (-0.13, 0.77)	0.43
Patch+N vs Patch 2H	1.00 (-0.16, 2.20)	0.10 (-0.89, 1.00)	0.43 (-0.29, 1.20)	0.22
Atr weekly vs Patch 2H	-0.21 (-0.89, 0.48)	0.30 (-0.82, 1.50)	-0.06 (-0.65, 0.51)	0.41
Binocular vs Patch 2H	-0.28 (-0.75, 0.19)	-0.02 (-0.93, 0.97)	-0.24 (-0.63, 0.19)	0.56
Patch 12H vs Patch 6H	-0.16 (-0.85, 0.53)	-0.41 (-1.5, 0.68)	-0.23 (-0.79, 0.33)	0.68
Atr daily vs Patch 6H	-0.32 (-1.10, 0.43)	-0.49 (-1.30, 0.33)	-0.39 (-0.93, 0.13)	0.74
Atr daily vs Patch 12H	-0.05 (-0.93, 0.87)	-0.28 (-1.20, 0.63)	-0.16 (-0.79, 0.46)	0.70
Atr weekly vs Atr daily	0.20 (-0.61, 1.00)	-0.31 (-1.40, 0.73)	0.02 (-0.61, 0.62)	0.41

Abbreviations: *H* hours per day, *Atr* atropine, *N* near activities, *D* distant activities, *Plano* plano lens over the sound eye
